# A comprehensive evaluation of the 8-gauge vacuum-assisted Mammotome^® ^system for ultrasound-guided diagnostic biopsy and selective excision of breast lesions

**DOI:** 10.1186/1477-7819-5-83

**Published:** 2007-07-30

**Authors:** Stephen P Povoski, Rafael E Jimenez

**Affiliations:** 1 Division of Surgical Oncology, Department of Surgery, Arthur G. James Cancer Hospital and Richard J. Solove Research Institute and Comprehensive Cancer Center, The Ohio State University, Columbus, Ohio, 43210, USA; 2 Department of Pathology, The Arthur G. James Cancer Hospital and Richard J. Solove Research Institute and Comprehensive Cancer Center, The Ohio State University, Columbus, Ohio, 43210, USA

## Abstract

**Background:**

Minimally invasive breast biopsy technology is now considered a standard of care for the diagnostic evaluation of suspicious breast lesions. The aim of the current study was to present a comprehensive evaluation of the 8-gauge vacuum-assisted Mammotome^® ^system for ultrasound-guided diagnostic biopsy and selective excision of breast lesions.

**Methods:**

A retrospective analysis was conducted of a series of 304 consecutive 8-gauge Mammotome^® ^procedures that were performed under ultrasound guidance by a single surgeon from March 2004 to December 2006. Multiple variables, including patient demographics, characteristics of the breast lesion (based on ultrasound and mammography), procedural and histopathology variables, and interval follow-up variables (based on ultrasound and mammography), were evaluated.

**Results:**

Among 304 procedures, 235 (77%) were performed with the presumption of complete excision of the ultrasound lesion during Mammotome^® ^core acquisition, while 69 (23%) were performed with only partial excision of the ultrasound lesion during Mammotome^® ^core acquisition (diagnostic tissue sampling only). 100% of all ultrasound lesions were accurately diagnosed, demonstrating no apparent false-negative results among the 256 patients that were compliant with follow-up at a median interval follow-up duration of 11 months (range 1 to 37). Likewise, 89% of all appropriately selected ultrasound lesions were completely excised, as demonstrated on interval follow-up ultrasound at a median time of 6 months (range, 3 to 16). There were no independent predictors of successful complete excision of any given appropriately selected ultrasound lesion by the ultrasound-guided 8-gauge Mammotome^® ^biopsy technique.

**Conclusion:**

The 8-gauge vacuum-assisted Mammotome^® ^system is highly accurate for ultrasound-guided diagnostic biopsy of suspicious breast lesions and is highly successful for complete excision of appropriately selected presumed benign breast lesions. This particular technology should be routinely offered to all appropriately selected patients that are evaluated by physicians involved in breast-specific health care.

## Background

Minimally invasive breast biopsy technology is now considered a standard of care for the diagnostic evaluation of suspicious breast lesions [1]. In this regard, an innovative vacuum-assisted breast biopsy technology was first commercially introduced in 1995 by Biopsys Medical, Inc. in Irvine, California [2] and was later acquired in 1997 and successfully marketed by Ethicon Endo-Surgery, Inc. in Cincinnati, Ohio [3]. This technology, the Mammotome^® ^breast biopsy system (Ethicon Endo-Surgery, Inc., Cincinnati, Ohio), was first utilized in stereotactic (mammographically-guided) applications [4] and was soon thereafter adapted for ultrasound-guided applications [5]. Since that time, multiple reports have been published using this technology for ultrasound-guided applications [6-33]. The majority of these reports have evaluated the 11-gauge Mammotome^® ^breast biopsy device [6-12,15,17-22,24-26,28-30]. To date, only eight reports evaluating the 8-gauge Mammotome^® ^breast biopsy device for ultrasound-guided applications have been published [13,14,16,23,27,31-33], with all such reports comparing the 8-gauge system to that of the 11-gauge system. However, no single report has comprehensively outlined the results of a large number of consecutively conducted ultrasound-guided breast biopsies performed by a single operator and solely using the 8-gauge Mammotome^® ^breast biopsy system. In the current report, we present a comprehensive evaluation of the 8-gauge vacuum-assisted Mammotome^® ^breast biopsy system for ultrasound-guided diagnostic biopsy and selective excision of 304 breast lesions.

## Methods

This study protocol was approved by the Clinical Scientific Review Committee and by the Cancer Institutional Review Board of the Arthur G. James Cancer Hospital and Richard J. Solove Research Institute and Comprehensive Cancer Center of the Ohio State University.

From the prospectively maintained operative log of a single surgeon (SPP), all patients who underwent either an ultrasound-guided diagnostic breast biopsy or an attempted ultrasound-guided excision of a breast lesion using the vacuum-assisted Mammotome^® ^breast biopsy system were identified. Prior to March 2004, both the 11-gauge Mammotome^® ^device and the 8-gauge Mammotome^® ^device were utilized by the above surgeon. Starting in March 2004, the 8-gauge Mammotome^® ^device was exclusively utilized. From March 2004 to December 2006, 304 consecutive procedures were performed by the above surgeon under ultrasound guidance using the 8-gauge Mammotome^® ^breast biopsy system. This particular group of 304 consecutive 8-gauge Mammotome^® ^procedures was utilized for the current study. As a point of reference, during the same time period, an additional 311 breast lesions were also biopsied by the same surgeon under ultrasound guidance using an Achieve^® ^automated, spring-loaded 14-gauge core biopsy device (Cardinal Health, Inc., McGraw Park, Illinois) or a Bard^® ^MaxCore™ automated, spring-loaded 14-gauge core biopsy device (C.R. Bard, Inc., Covington, Georgia). All these breast biopsy procedures were performed at JamesCare in Dublin (Dublin, Ohio), our comprehensive breast health services facility of the Arthur G. James Cancer Hospital and Richard J. Solove Research Institute and Comprehensive Cancer Center of the Ohio State University.

All breast lesions evaluated by the 8-gauge Mammotome^® ^breast biopsy system were sonographically visible and were classified as BI-RADS (American College of Radiology Breast Imaging Reporting and Data System) category 3, 4, or 5.

All 8-gauge Mammotome^® ^procedures were performed by a single surgeon (SPP) in the ultrasonography suite at JamesCare in Dublin with the patient positioned supine on a Biopsys exam table (model 106-004, Biopsys Medical, Inc., Irvine, California). Freehand real-time ultrasound guidance was performed using high-resolution linear array transducers. Initially, ultrasound guidance was performed using a Philips HDI 5000 SonoCT system (Philips Medical Systems Andover, Massachusetts), with a variable frequency transducer L12-5 (range 4.75 to 10.0 MHz). Later on, ultrasound guidance was performed using a Philips iU22 system (Philips Medical Systems Andover, Massachusetts), with either a variable frequency transducer L12-5 (range 4.75 to 10.0 MHz) or a variable frequency transducer L17-5 (range 5.75 to 7.0 MHz). Local anesthetic, consisting of 1% lidocaine plain (used for the skin and superficial tissues, and ranging from 8 to 12 mL) and 1% lidocaine containing 1:100,000 mixture of epinephrine (used for the deeper breast tissues surround the ultrasound lesion, and ranging from 15 to 25 mL) was utilized. After local anesthetic was administered, a 5 mm skin incision was made with a #11 blade and through which the 8-gauge Mammotome^® ^device was passed and positioned just beneath the sonographic lesion using real-time ultrasound guidance. Then, multiple 8-gauge Mammotome^® ^cores were sequentially taken under real-time ultrasound guidance. In cases in which an attempt was made to completely excise the ultrasound lesion during core acquisition, multiple cores were taken under ultrasound guidance while sequentially rotating the 8-gauge Mammotome^® ^device over an array spanning approximately 180 degrees. The number of Mammotome^® ^cores harvested and the surgeon's impression as to whether the ultrasound lesion was only partially sampled for diagnostic tissue sampling purposes only or whether the ultrasound lesion appeared to be completely excised was documented in the operative report. An attempt at complete ultrasound lesion excision was assessed in real-time by taking longitudinal and transverse ultrasound images both during core acquisition and after the completion of core acquisition. After the completion of core acquisition, the 8-gauge Mammotome^® ^device was removed and a 14-gauge Cormark™ rigid microclip device (Ethicon Endo-Surgery, Inc., Cincinnati, Ohio) was inserted under ultrasound guidance through the same breast parenchymal track for placement of a microclip into the residual ultrasound lesion of a partial removed lesion or into the biopsy cavity of a presumed completely removed lesion. Thereafter, manual compression to the breast was performed for approximately ten minutes to assure adequate hemostasis. The 5 mm incision site was then generally closed with Steri-Strip™ adhesive skin closures (3 M Health Care, St. Paul, Minnesota).

For all patients, the findings on pathologic evaluation of the 8-gauge Mammotome^® ^breast biopsy were first discussed by telephone with the patient at the soonest availability of those results. All patients with abnormal findings on pathologic evaluation that warranted surgical intervention were appropriately managed. All patients with benign findings on pathologic evaluation were asked to return to JamesCare in Dublin for interval follow-up clinical examination and breast imaging (generally both ultrasound and mammography) at approximately six months after the initial 8-gauge Mammotome^® ^procedure. Some variability in timing of interval follow-up for patients with benign pathology occurred secondary to patient availability and patient compliance issues. Some patients with benign pathology remained noncompliant and had no interval follow-up, even after multiple attempts to arrange interval follow-up for such patients.

The data collection of all variables for this study was accomplished by way of retrospective review of the electronic medical records system of the Ohio State University Medical Center. Multiple variables were assessed and evaluated, including patient demographics, characteristics of the breast lesion (based on ultrasound and mammography), procedural and histopathology variables for the 8-gauge Mammotome^® ^biopsy technique, and interval follow-up variables (based on ultrasound and mammography).

The three dimensions (length, width, and height) of each ultrasound lesion were obtained from the official ultrasound report. The length, width, and height were then arbitrarily assigned to the designation of dimension 1, dimension 2, or dimension 3, based solely on their descending order in size. The volume of the original ultrasound lesion and the volume of any residual ultrasound lesion seen at interval follow-up were both calculated using the formula of the volume of an ellipsoid [4/3·π·length axis radius·width axis radius·height axis radius], rather than using the formula of a cuboid [length·width·height], since the three-dimensional shape of any given ultrasound lesion generally better approximated that of an ellipsoid rather than that of a cuboid [34]. The three dimensions (length, width, and height) of the 8-gauge Mammotome^® ^cores harvested at the time of each procedure were obtained from the official pathology report. The estimated volume of the 8-gauge Mammotome^® ^cores harvested was calculated using the formula of the volume of a cuboid rather than the formula of the volume of an ellipsoid, since the 8-gauge Mammotome^® ^cores harvested were generally measured by simply placing them side-by-side into a configuration resembling a cuboid.

The software program SPSS 14.0 for Windows (SPSS, Inc., Chicago, Illinois) was used for all statistical analyses. For univariate comparisons of categorical variables, either Pearson chi-square test or Fisher exact test was utilized. Continuous variables were expressed as median (range). For univariate comparisons of continuous variables, one-way analysis of variance (ANOVA) was utilized. All reported univariate P-values were two-sided. All univariate P-values determined to be 0.05 or less were considered to be significant. Multivariate logistic regression analysis was performed in appropriately selected situations on all variables with a univariate P-value of 0.10 or less, in order to assess for the determination of possible independent predictors.

## Results

Patient demographics and characteristics of the original breast lesions seen on the pre-Mammotome^® ^procedure ultrasound are shown in Table 1. Of the 304 ultrasound-guided 8-gauge Mammotome^® ^biopsy procedures performed, 235 (77%) were performed with the presumption that the ultrasound lesion had been completely excised during Mammotome^® ^core acquisition, while 69 (23%) were performed with only partial excision of the ultrasound lesion during Mammotome^® ^core acquisition (diagnostic tissue sampling only). Patients in which the ultrasound lesion appeared to be completely excised during Mammotome^® ^core acquisition were generally younger, had a predilection toward BI-RADS category 3 and 4 classification of the breast lesion, and had a significantly smaller original breast lesion size (including all three dimensions of the original ultrasound lesion and the original ultrasound lesion volume) based on the pre-Mammotome^® ^procedure ultrasound findings. Whereas, patients in which only diagnostic tissue sampling was undertaken during Mammotome^® ^core acquisition were generally older, had a predilection toward BI-RADS category 4 and 5 classification of the breast lesion, and had a significantly larger original breast lesion size based on the pre-Mammotome^® ^procedure ultrasound findings.

**Table 1:** Patient demographics and characteristics of the original breast lesions seen on the pre-Mammotome^® ^procedure ultrasound: For all cases (n = 304), for cases in which an attempt to completely excise the breast lesion was made (n = 235), and for cases in which only diagnostic tissue sampling of the breast lesion was undertaken (n = 69)

	All cases (n = 304)	Attempted lesion excision (n = 235)	Only diagnostic lesion sampling (n = 69)	P-value
Age (years)	47 (18–87)	44 (18–87)	53 (22–84)	< 0.001
Gender				0.539
Female	301 (99%)	233 (99%)	68 (99%)	
Male	3 (1%)	2 (1%)	1 (1%)	
Breast				0.158
Right	136 (45%)	100 (43%)	36 (52%)	
Left	168 (55%)	135 (57%)	33 (48%)	
Palpable tumor				0.057
Yes	150 (49%)	109 (46%)	41 (59%)	
No	154 (51%)	126 (54%)	28 (41%)	
Lesion location				0.185
UOQ	160 (53%)	124 (53%)	36 (52%)	
UIQ	63 (21%)	45 (19%)	18 (26%)	
LOQ	50 (16%)	44 (19%)	6 (9%)	
LIQ	24 (8%)	18 (8%)	6 (9%)	
Subareolar	7 (2%)	4 (2%)	3 (4%)	
BI-RADS classification of ultrasound				< 0.001
Category 3	55 (18%)	49 (21%)	6 (9%)	
Category 4	231 (76%)	179 (76%)	52 (75%)	
Category 5	18 (6%)	7 (3%)	11 (16%)	
Was the ultrasound lesion also visible on pre-procedural mammogram?				0.251
Yes	190 (63%)	141 (60%)	49 (71%)	
No	58 (19%)	48 (20%)	10 (15%)	
Pre-procedural mammogram not done	56 (18%)	46 (20%)	10 (15%)	
Original ultrasound lesion measurements				
Dimension 1 (cm)	1.14 (0.33–5.53)	1.10 (0.33–3.12)	1.24 (0.50–5.53)	0.004
Dimension 2 (cm)	0.99 (0.24–3.74)	0.95 (0.24–2.52)	1.01 (0.40–3.74)	0.013
Dimension 3 (cm)	0.66 (0.22–1.80)	0.62 (0.22–1.70)	0.87 (0.30–1.80)	< 0.001
Volume of lesion (cubic cm)	0.38 (0.01–9.64)	0.35 (0.01–4.36)	0.63 (0.04–9.64)	< 0.001

UOQ, upper outer quadrant; LOQ, lower outer quadrant; UIQ, upper inner quadrant; LIQ, lower inner quadrant; BI-RADS, breast imaging reporting and data system

Procedural and histopathology variables for the ultrasound-guided 8-gauge Mammotome^® ^biopsy procedures are shown in Table 2. For those cases in which the ultrasound lesion appeared to be completely excised during Mammotome^® ^core acquisition, significantly more tissue was removed (based on the estimated number of Mammotome^® ^core removed, estimated volume of Mammotome^® ^cores removed, and percentage of estimated volume of Mammotome^® ^cores removed as compared to the volume of the original breast lesion calculated on the pre-Mammotome^® ^procedure ultrasound) and the final histopathology more frequently rendered a benign diagnosis (predominately fibroadenomas). For those cases in which only diagnostic tissue sampling was undertaken during Mammotome^® ^core acquisition, significantly less tissue was removed and the final histopathology more frequently rendered a malignant diagnosis (predominately invasive breast carcinomas). The overall number of post-procedural complications (which included hematoma formation, skin ecchymosis, or both) and the severity of such complications seen were not significantly different between the two groups. No intraoperative surgical management of any post-procedural complications was ever required. No post-procedural infectious complications were encountered.

**Table 2:** Procedural and histopathology variables for the ultrasound-guided 8-gauge Mammotome^® ^biopsy technique: For all cases (n = 304), for cases in which an attempt to completely excise the breast lesion was made (n = 235), and for cases in which only diagnostic tissue sampling of the breast lesion was undertaken (n = 69)

	All cases (n = 304)	Attempted lesion excision (n = 235)	Only diagnostic lesion sampling (n = 69)	P-value
Estimated number of Mammotome^® ^cores removed	8 (2–38)	9 (3–38)	6 (2–17)	< 0.001
Estimated volume of Mammotome^® ^cores removed (cubic cm)	2.18 (0.26–37.13)	2.50 (0.60–37.13)	1.38 (0.26–4.38)	< 0.001
Percentage of estimated volume of Mammotome^® ^cores removed as compared to volume of original ultrasound lesion	615% (9%–18,086%)	730% (113%–18,086%)	222% (9%–2263%)	< 0.001
Lesion histopathology				< 0.001
Fibroadenoma	126 (41%)	112 (48%)	14 (20%)	
Other benign findings	107 (35%)	88 (37%)	19 (28%)	
Benign tumors	10 (3%)	7 (3%)	3 (4%)	
Invasive breast cancer	47 (16%)	20 (9%)	27 (39%)	
DCIS	4 (1%)	2 (1%)	2 (3%)	
ICPC	2 (1%)	1 (< 0.5%)	1 (1%)	
Lymphoma	3 (1%)	1 (< 0.5%)	2 (3%)	
SQBCC	1 (< 0.5%)	0 (0%)	1 (1%)	
Epithelial atypia	4 (1%)	4 (2%)	0 (0%)	
Post-procedural complications (hematoma formation, skin ecchymosis, or both)	24 (8%)	20 (9%)	4 (6%)	0.462

DCIS, ductal carcinoma in situ; ICPC, intracystic papillary carcinoma; SQBCC, subcutaneous basal cell carcinoma

Interval follow-up variables are shown in Table 3. Of the 304 patients who underwent an ultrasound-guided 8-gauge Mammotome^® ^biopsy procedure, 256 (84%) patients were compliant and returned for interval follow-up, while 48 (16%) were noncompliant and did not returned for interval follow-up. The frequency of such interval follow-up was identical both for those patients in which the ultrasound lesion appeared to be completely excised during Mammotome^® ^core acquisition and for those patients in which only diagnostic tissue sampling was undertaken during Mammotome^® ^core acquisition. Interval follow-up breast imaging was performed in 189 (62%) patients at a median time of 6 months (range, 3 to 16). This included 188 (62%) patients who underwent interval follow-up ultrasound and 179 (59%) patients who underwent interval follow-up mammogram. Instead of interval follow-up breast imaging, 64 (21%) of the patients underwent some sort of subsequent excisional breast procedure at a time shortly after their initial ultrasound-guided 8-gauge Mammotome^® ^biopsy procedure. This included 46 invasive breast carcinomas, 4 cases of ductal carcinoma in situ, 2 intracystic papillary carcinomas, 1 subcutaneous basal cell carcinoma of the breast, 2 cases of epithelial atypia, 3 benign breast tumors requiring complete excision, 4 benign breast findings that were incidentally excised within the same excisional specimen as a known breast carcinoma, and 2 cases in which a benign finding was incidentally excised as part a planned prophylactic mastectomy. Far fewer patients in the group in which only diagnostic tissue sampling was undertaken during Mammotome^® ^core acquisition had interval follow-up breast imaging since far more of those same patients had a malignant breast lesion that required some sort of subsequent excisional breast procedure. For those patients with a breast malignancy, 39 of 53 (74%) underwent subsequent successful breast conservation therapy, while 14 of 53 (26%) ultimately required a mastectomy (including 5 for multifocal/multicentric disease, 3 for a failed attempt at breast conservation therapy, 3 for subareolar tumor location, and 3 as patient choice).

**Table 3:** Interval follow-up variables: For all cases (n = 304), for cases in which an attempt to completely excise the breast lesion was made (n = 235), and for cases in which only diagnostic tissue sampling of the breast lesion was undertaken (n = 69)

	All cases (n = 304)	Attempted lesion excision (n = 235)	Only diagnostic lesion sampling (n = 69)	P-value
Was any IFU done by patient?				0.678
Yes	256 (84%)	199 (85%)	57 (83%)	
No	48 (16%)	36 (15%)	12 (17%)	
Was IFU imaging performed?				<0.001
Yes	189 (62%)	170 (72%)	19 (28%)	
No	51 (17%)	38 (16%)	13 (19%)	
Instead had subsequent excisional breast procedure performed	64 (21%)	27 (12%)	37 (54%)	
Median time to IFU imaging (months) for those (n = 189) undergoing IFU imaging	6 (3–16)	6 (3–16)	6 (3–8)	0.110
Was IFU ultrasound performed?				<0.001
Yes	188 (62%)	170 (72%)	18 (26%)	
No	52 (17%)	38 (16%)	14 (20%)	
Instead had subsequent excisional breast procedure performed	64 (21%)	27 (12%)	37 (54%)	
Was a residual ultrasound lesion visible for those undergoing IFU ultrasound (n = 188)?				<0.001
Yes	34 (18%)	19 (11%)	15 (83%)	
No	154 (82%)	151 (89%)	3 (17%)	
Was IFU mammogram performed?				<0.001
Yes	179 (59%)	162 (69%)	17 (25%)	
No	61 (20%)	46 (20%)	15 (22%)	
Instead had subsequent excisional breast procedure done	64 (21%)	27 (12%)	37 (54%)	
Was a residual mammographic lesion visible for those undergoing IFU mammogram (n = 179)?				0.001
Yes	11 (6%)	6 (4%)	5 (29%)	
No	168 (94%)	156 (96%)	12 (71%)	
For those undergoing a pre-procedural mammogram (which demonstrated a mammographic lesion) and who had both an IFU ultrasound and an IFU mammogram, was a residual ultrasound lesion visible on IFU ultrasound (n = 110)?				<0.001
Yes	21 (19%)	14 (14%)	7 (88%)	
No	89 (81%)	88 (86%)	1 (12%)	
For those undergoing a pre-procedural mammogram (which demonstrated a mammographic lesion) and who had both an IFU ultrasound and an IFU mammogram, was a residual mammographic lesion visible on IFU mammogram (n = 110)?				0.001
Yes	9 (8%)	5 (5%)	4 (50%)	
No	101 (92%)	97 (95%)	4 (50%)	
Median duration of IFU (months) for those (n = 256) undergoing IFU	11 (1–37)	9 (2–37)	13 (1–30)	0.146

IFU, Interval follow-up

As is shown in Table 3, of those patients who were compliant and returned for interval follow-up ultrasound imaging (n = 188), 154 (82%) had no residual ultrasound lesion visible on the interval follow-up ultrasound. As would be expected, significantly more patients in which the ultrasound lesion appeared to be completely excised during Mammotome^® ^core acquisition had no residual ultrasound lesion visible on the interval follow-up ultrasound (n = 151/170, 89%), as compared to patients in which the procedure was performed for the purpose of only diagnostic tissue sampling during Mammotome^® ^core acquisition (n = 3/18, 17%). Thus, complete ultrasound lesions removal was confirmed on interval follow-up ultrasound in 89% of patients in which the ultrasound lesion appeared to be completely excised during Mammotome^® ^core acquisition. Overall, 34 (18%) patients who underwent an ultrasound-guided 8-gauge Mammotome^® ^biopsy procedure and who had interval follow-up ultrasound demonstrated a residual ultrasound lesion on the interval follow-up ultrasound, consisting of 19 (11%) of those in which an attempt was made to completely excise the original ultrasound breast lesion and 15 (83%) of those in which the procedure was performed for the purpose of only diagnostic tissue sampling.

Residual ultrasound lesion characteristics seen on interval follow-up ultrasound imaging are shown in Table 4. All residual ultrasound lesion characteristics seen on interval follow-up ultrasound imaging (including all three dimensions of the residual ultrasound lesion, the residual ultrasound lesion volume, and the percentage of volume of the residual ultrasound lesion as compared to the original ultrasound lesion) were significantly less for those 19 cases in which an attempt was made to completely excise the original ultrasound breast lesion as compared to those 15 cases in which only diagnostic tissue sampling was performed.

**Table 4:** Residual ultrasound lesion characteristics seen on interval follow-up ultrasound imaging (n = 188): For all cases (n = 34), for cases in which an attempt to completely excise the breast lesion was made (n = 19), and for cases in which only diagnostic tissue sampling of the breast lesion was undertaken (n = 15)

	All cases (n = 34)	Attempted lesion excision (n = 19)	Only diagnostic lesion sampling (n = 15)	P-value
Residual ultrasound lesion measurements				
Dimension 1 (cm)	0.95 (0.32–2.50)	0.79 (0.46–1.70)	1.37 (0.32–2.50)	0.003
Dimension 2 (cm)	0.74 (0.29–2.03)	0.70 (0.42–1.50)	1.02 (0.29–2.03)	0.022
Dimension 3 (cm)	0.53 (0.26–1.50)	0.44 (0.26–0.81)	0.86 (0.29–1.50)	< 0.001
Volume of lesion (cubic cm)	0.18 (0.01–3.93)	0.13 (0.03–1.07)	0.65 (0.01–3.93)	0.006
Percentage of volume of residual ultrasound lesionas compared to volume of original ultrasound lesion	33% (3%–366%)	22% (3%–78%)	84% (12%–366%)	0.002

In Table 3, the presence or absence of a residual mammographic lesion on interval follow-up imaging poorly correlated with the presence or absence of a residual ultrasound lesion on interval follow-up imaging. This was best exemplified by the fact that within the group of patients that underwent a pre-procedural mammogram (which demonstrated a mammographic lesion) and had both an interval follow-up ultrasound and interval follow-up mammogram that while 7 of 8 (88%) patients undergoing diagnostic tissue sampling only during Mammotome^® ^core acquisition had a residual ultrasound lesion on interval follow-up, comparatively only 4 of 8 (50%) of the same such patients had a residual mammographic lesion on interval follow-up.

As is shown in Table 5, for cases in which an attempt was made to completely excise the ultrasound breast lesion, the ability to originally palpate the ultrasound breast lesion prior to the 8-gauge Mammotome^® ^biopsy procedure did not significantly influence whether a residual ultrasound lesion could be seen on interval follow-up ultrasound imaging. However, and as would be intuitively deducted, the median size of the original ultrasound breast lesion (including all three dimensions of the lesion and the lesion volume) significantly correlated with whether a residual ultrasound lesion could be seen on interval follow-up ultrasound imaging, with smaller such original ultrasound breast lesions being seen in the group having no residual ultrasound lesion seen on interval follow-up ultrasound imaging and with larger such original ultrasound breast lesions being seen in the group having a residual ultrasound lesion seen on interval follow-up ultrasound imaging. Despite this apparent significant size correlation that was demonstrated on ANOVA, multivariate analysis using logistic regression failed to show that any single original ultrasound breast lesion size parameter could independently predict ones ability to know whether a residual ultrasound lesion would be seen on interval follow-up ultrasound imaging.

**Table 5:** Variables influencing whether a residual ultrasound lesion is seen on interval follow-up ultrasound imaging for cases in which an attempt was originally made to completely excise the breast lesion and in which interval follow-up ultrasound imaging was obtained (n = 170)

	All cases (n = 170)	No residual ultrasound lesion seen (n = 151)	Residual ultrasound lesion seen (n = 19)	P-value
Was the original breast lesion palpable				0.242
Yes	77 (45%)	66 (44%)	11 (58%)	
No	93 (55%)	85 (56%)	8 (42%)	
Original ultrasound lesion measurements				
Dimension 1 (cm)	1.13 (0.37–3.12)	1.10 (0.37–2.48)	1.44 (0.90–3.12)	< 0.001
Dimension 2 (cm)	0.98 (0.24–2.52)	0.93 (0.24–1.96)	1.32 (0.69–2.52)	< 0.001
Dimension 3 (cm)	0.63 (0.22–1.50)	0.60 (0.22–1.50)	0.85 (0.53–1.17)	0.007
Volume of lesion (cubic cm)	0.35 (0.01–4.36)	0.33 (0.01–3.48)	0.83 (0.23–4.36)	< 0.001

To date, the median duration of interval follow-up for those 256 patients who were compliant and who returned for interval follow-up has been 11 months (range, 1 to 37) (Table 3). Of those 189 patients who have had interval follow-up imaging, only 4 (2%) patients have developed a subsequent clinical or breast imaging finding within the same location of the breast as where the original ultrasound-guided 8-gauge Mammotome^® ^biopsy was performed that has subsequently required an additional breast biopsy procedure. These have included three repeat ultrasound-guided 8-gauge Mammotome^® ^biopsy procedures (at 9, 12, and 30 months after the original Mammotome^® ^biopsy procedure) and one excisional biopsy (at 5 months after the original Mammotome^® ^biopsy procedure). All four such repeat breast biopsy procedures simply showed benign findings that were consistent with the results of their previous original Mammotome^® ^biopsy findings. Therefore, to date, there have been no instances within the group of 256 patients that were compliant with interval follow-up in which the histopathology results of the original ultrasound-guided 8-gauge Mammotome^® ^biopsy have changed (i.e, there have been no false-negative results).

## Discussion

Within the last 15 years, the diagnostic evaluation of suspicious breast lesions has been significantly impacted upon by the development of innovative minimally invasive breast biopsy technologies. Such practices have been rapidly adopted by those physicians involved in breast-specific health care and are now routinely considered a standard of care [1]. To date, only eight published reports (Table 6) can be found in the literature [13,14,16,23,27,31-33] that have formally evaluated the 8-gauge vacuum-assisted Mammotome^® ^breast biopsy device for ultrasound-guided applications, with all such reports comparing the 8-gauge system to that of the 11-gauge system. However, no single report has comprehensively detailed the results of a large number of consecutively conducted ultrasound-guided breast biopsies performed by a single operator and solely using the 8-gauge Mammotome^® ^breast biopsy system. In that specific regard, the current study represents a comprehensive evaluation of the 8-gauge vacuum-assisted Mammotome^® ^system for ultrasound-guided diagnostic biopsy and selective excision of breast lesions.

**Table 6:** All studies reporting experience with the ultrasound-guided 8-gauge vacuum-assisted Mammotome^® ^biopsy technique

Authors	Year	Total number of procedures	8-Gauge	11-Gauge	Performed by	Stated method of interval follow-up (IFU)
Fine et al. [13]	2002	124	75	49	≥ 5 physicians	ultrasound and clinical exam at 6 months
Johnson et al. [14]	2002	101	not specified	not specified	not specified	mammography at 6 months
Fine et al. [16 ]	2003	216	127	89	≥ 5 physicians	ultrasound and clinical exam at 6 months
Carpentier et al. [23]	2005	42	24	18	2 physicians	ultrasound at 8 weeks, 6 months, and 12 months
Grady et al. [27]	2005	542	189^#^	353^#^	2 physicians	surgical excision in 47 of 52 patients evaluated for atypia
Sebag et al. [31]	2006	650	230*	420*	3 centers	ultrasound, mammography, and clinical exam at 6 and 12 months
Vargos et al. [32]	2006	210	169	41	not specified	clinical exam at time not specified
Xiao et al. [33]	2006	174	104^§^	70^§^	2 physicians	not specified
Povoski	2007	304	304	0	1 physician	ultrasound and mammography at a median IFU of 6 months

IFU, Interval follow-up

^# ^Via a personal e-mail communication with Dr. Ian Grady (Redding, California) from April 23, 2007, it was estimated and verified by Dr. Grady that approximately 50% (approximately 189) of the 378 cases performed after July 2002 where done by the ultrasound-guided 8-gauge Mammotome^® ^biopsy approach and approximately 50% (approximately 189) of the 378 cases performed after July 2002 where done by the ultrasound-guided 11-gauge Mammotome^® ^biopsy approach. Therefore, including the 164 cases that were done by the ultrasound-guided 11-gauge Mammotome^® ^biopsy approach before July 2002, it was estimated that overall a total of approximately 353 ultrasound-guided 11-gauge Mammotome^® ^biopsy procedures were performed and approximately 189 ultrasound-guided 8-gauge Mammotome^® ^biopsy procedures were performed.

* Via a personal e-mail communication with Dr. Philippe Sebag (Lyon, France) from January 23, 2007, it was estimated and verified by Dr. Sebag that approximately 35% (approximately 230) of the 650 cases performed where done by the ultrasound-guided 8-gauge Mammotome^® ^biopsy approach and approximately 65% (approximately 420) of the 650 cases performed were done by the ultrasound-guided 11-gauge Mammotome^® ^biopsy approach.

^§ ^Via a personal e-mail communication with Dr. Li Xiao (Changsha, China) from May 16, 2007, it was estimated and verified by Dr. Xiao that approximately 60% (approximately 104) of the 174 cases performed where done by the ultrasound-guided 8-gauge Mammotome^® ^biopsy approach and approximately 40% (approximately 70) of the 174 cases performed were done by the ultrasound-guided 11-gauge Mammotome^® ^biopsy approach.

In the current study, 100% of all ultrasound lesions were accurately diagnosed by the ultrasound-guided 8-gauge Mammotome^® ^biopsy technique, demonstrating no apparent false-negative results among the 256 patients that were compliant with follow-up at a current median interval follow-up duration of 11 months (range 1 to 37). In this regard, since over 50% of the invasive breast cancers diagnosed in the present series were less than one centimeter in size, it is very apparent that the ultrasound-guided 8-gauge Mammotome^® ^biopsy technique is highly advantageous for allowing generous and representative tissue sampling and for high accuracy of correctly diagnosing suspicious small subcentimeter breast lesions. In a similar regard, the ultrasound-guided 8-gauge Mammotome^® ^biopsy device may potentially minimize the risk of histopathologic underestimation and false negative results [25] that could potentially be associated with the use of an automated, spring-loaded 14-gauge core biopsy device. Such an event could theoretically occur in a similar group of suspicious small subcentimeter breast lesions secondary to positional overshooting or undershooting of the automated, spring-loaded core acquisition chamber while firing the 14-gauge core biopsy device.

In the current study, 89% of all appropriately selected ultrasound lesions were completely excised by the ultrasound-guided 8-gauge Mammotome^® ^biopsy technique, as demonstrated on interval follow-up ultrasound at a median time of 6 months (range, 3 to 16). This figure of a nearly 90% success of complete excision on ultrasound-guided 8-gauge Mammotome^® ^biopsy exceeds the 73% success of complete excision previously reported by Fine et al [16]. The greater success of excision in the current series is most likely attributed to several factors, including the larger mean ultrasound lesion size reported by Fine et al [16] and the exclusive use of the 8-gauge Mammotome^® ^device in the current series versus the use of both 8-gauge and 11-gauge Mammotome^® ^devices in the Fine et al series [16]. Likewise, the ultrasound-guided 8-gauge Mammotome^® ^biopsy technique allows for on-going, real-time, ultrasound-guided "tailoring" of the excision cavity of any given presumed benign lesion that the operator is attempting to completely excise at the time of the Mammotome^® ^procedure. This is itself a potential advantage over that of any percutaneous en bloc excision technique, since such percutaneous en bloc excision techniques generally allow for only a one-time attempt at tissue acquisition and can theoretically require an additional diagnostic procedure for complete excision any given presumed benign lesion if positioning variation results in altered coordinates of the region of initial en bloc tissue acquisition.

Therefore, when a breast lesion is sonographically visible, it is our opinion that the 8-gauge Mammotome^® ^system is the desired and optimal method both for accurate diagnosis of any given suspicious ultrasound lesion and for complete removal of appropriately selected presumed benign ultrasound lesions. In this regard, and as emphasized by Sebag et al [31], the 8-gauge Mammotome^® ^system allows for more rapid and larger volume tissue acquisition, resulting in more representative tissue sampling in cases of diagnostic breast biopsy and need for removal of fewer total Mammotome^® ^cores in cases of attempted complete ultrasound lesion excision. Furthermore, as do all surgeons, we strongly advocate the adherence to sound oncologic principles for the complete surgical excision of malignant breast lesions.

Since multivariate analysis using logistic regression failed to show that any single original ultrasound breast lesion parameter could independently predict the ability to know whether a residual ultrasound breast lesion would be seen on interval follow-up ultrasound imaging, then one can not inexplicitly recommend a particular breast lesion size threshold below which any given ultrasound breast lesion can be successfully and completely excised by the ultrasound-guided 8-gauge Mammotome^® ^biopsy technique. Despite this lack of any exact evidence to allow one to set a finite size criteria for the successful complete excision of appropriately selected ultrasound breast lesions by the ultrasound-guided 8-gauge Mammotome^® ^biopsy technique, the present authors arbitrarily recommend limiting any attempts at complete excision to that of such breast lesions which approximate a prolate ellipsoid (i.e., cigar-shaped) or a scalene ellipsoid (i.e., three unequal dimensions) and to that of such breast lesions that have a maximum lesion length of 2.5 cm and maximum lesion width of 1.5 cm. In contrast, no upper limit for the maximum lesion height seems necessary when the attempted ultrasound-guided 8-gauge Mammotome^® ^excision procedure is performed by appropriate placement of the Mammotome^® ^device immediately beneath the ultrasound lesion. However, the most difficult breast lesions to excise are those breast lesions that approximate an oblate ellipsoid (i.e., disk-shaped) in which the largest two nearly identical dimensions exceeds the 2.5 cm maximum size limit or those breast lesions that approximate a perfect sphere in which the diameter exceeds the 2.5 cm maximum size limit. These above recommendations are partially based on the experience of the present surgeon (SPP) in the currently reported series, as well as are based on the actual length of the tissue collection chamber (23 mm) and the actual inner diameter of the cutter blade (3.9 mm) for the 8-gauge Mammotome^® ^biopsy device [35].

A major shortcoming of two of the other previously reported ultrasound-guided 8-gauge Mammotome^® ^biopsy studies (Table 6) has been their reliance solely upon interval follow-up mammography or upon interval follow-up clinical examination to assess the breast for the success of attempted lesion excision and their apparent lack of the use of interval follow-up ultrasound [14,32]. In the study by Johnson et al [14], they used only mammography at the time of six-month interval follow-up. They reported that there were no cases in which a documented abnormal pre-procedural mammogram and subsequent six-month mammogram demonstrated a retained lesion. In the current study, the analogous observation seems quite different as compared to Johnson et al [14]. As is clearly shown in Table 3, for all patients with a pre-procedural mammographic abnormality that underwent only diagnostic tissue sampling during Mammotome^® ^core acquisition, while 88% had a residual ultrasound lesion on interval follow-up ultrasound, only 50% had a residual mammographic lesion on interval follow-up mammography. This clearly demonstrates that the presence or absence of a residual mammographic lesion at the time of interval follow-up poorly correlates to the presence or absence of a corresponding residual ultrasound lesion. Similarly, in the study by Vargas et al [32], no specific interval follow-up imaging was reported and only interval follow-up clinical examination was discussed. For similar reasons, and as is reported in the current study, since less than one-half of our ultrasound lesions that were attempted to be excised at the time of the ultrasound-guided 8-gauge Mammotome^® ^biopsy were originally palpable, then interval follow-up clinical examination alone would seem to be a poor predictor of the success of attempted lesion excision. In this regard, we highly recommend interval follow-up ultrasound surveillance of the area of the previous ultrasound-guided 8-gauge Mammotome^® ^biopsy at approximately six months after such a biopsy, as well as highly recommend consideration of placement of a microclip into the original biopsy site at the time of the previous ultrasound-guided 8-gauge Mammotome^® ^biopsy as a mechanism for aiding in the ongoing mammographic surveillance and/or future mammographic surveillance of such patients at an appropriately selected age. The importance for interval follow-up ultrasound surveillance is clearly demonstrated in the present series, and has been previously recognized by the authors of several of the other ultrasound-guided 8-gauge Mammotome^® ^biopsy papers [13,16,23,31].

Lastly, the importance of placement of a microclip into the biopsy site at the time of the original Mammotome^® ^biopsy procedure goes far beyond its potential usefulness for ongoing and/or future mammographic surveillance and clearly extends into the realm of its usefulness when one is faced with the mandatory need for subsequent imaged-guided excision to an area demonstrating malignancy that was previously either partially excised or completely excised during the original Mammotome^® ^biopsy procedure. In such situations, the previous placement of a microclip into the biopsy site at the time of the original Mammotome^® ^biopsy procedure allows for potential flexibility for subsequent image-guided excision, especially when a persistent ultrasound lesion is not well visualized on post-Mammotome^® ^biopsy ultrasound imaging. Some have argued against microclip placement during ultrasound-guided Mammotome^® ^biopsy secondary to the concept of hematoma-directed ultrasound-guided excision [36,37]. While this technique has proven usefulness, its major drawback is the time-dependent limitation of this technique secondary to the gradual reabsorption of the hematoma [37]. Therefore, microclip placement into the biopsy site at the time of the original Mammotome^® ^biopsy procedure remains critical even for those that advocate hematoma-directed ultrasound-guided excision since image-guided excision of the site of the original tumor can still be directed toward localization of the microclip in cases in which rapid hematoma resorption has taken place. Along similar lines, microclip placement into the biopsy site at the time of the original Mammotome^® ^biopsy procedure can be critical in those instances in which preoperative neoadjuvant chemotherapy is being considered, since a 100% pathologic response from preoperative neoadjuvant chemotherapy may result in complete resolution of any detectable mammographic or ultrasound lesion at the site of the original tumor [38-40]. Once again, in this situation, image-guided excision of the site of the original tumor can still be directed toward localization of the microclip.

## Conclusion

The 8-gauge vacuum-assisted Mammotome^® ^system is highly accurate for ultrasound-guided diagnostic biopsy of suspicious breast lesions and is highly successful for ultrasound-guided complete removal of appropriately selected presumed benign breast lesions. This particular technology is a very viable and appropriate alternative to smaller-gauged, automated, spring-loaded core biopsy and to diagnostic open surgical breast biopsy for maximizing the accuracy of and for minimizing the invasiveness of any given diagnostic breast biopsy procedure. In this regard, we believe that the 8-gauge vacuum-assisted Mammotome^® ^biopsy system should be routinely offered to all appropriately selected patients that are evaluated by physicians involved in breast-specific health care.

## Abbreviations

UOQ, upper outer quadrant; LOQ, lower outer quadrant; UIQ, upper inner quadrant; LIQ, lower inner quadrant; BI-RADS, breast imaging reporting and data system; DCIS, ductal carcinoma in situ; ICPC, intracystic papillary carcinoma; SQBCC, subcutaneous basal cell carcinoma; IFU, interval follow-up

## Competing interests

The author(s) declare that they have no competing interests.

## Authors' contributions

SPP was the surgeon who performed all the ultrasound-guided diagnostic biopsy and selective excision of breast lesions using the 8-gauge vacuum-assisted Mammotome^® ^system. He designed the current study, collected the data, and performed all data analyses. He organized, wrote, and edited all aspects of this manuscript. He approved the final version of this manuscript. REJ was the pathologist who was involved in reading the histopathology for many of the cases contained within this series. He edited all aspects of this manuscript and approved the final version of this manuscript.

## References

[B1] Burak WE, Agnese DM, Povoski SP (2004). Advances in the surgical management of early stage invasive breast cancer. Curr Probl Surg.

[B2] SEC Info: Quarterly Report of Biopsys Medical, Inc·Form 10-Q·For 9/30/96. http://www.secinfo.com/dRqWm.9XXu.htm.

[B3] PRNewswire: Johnson & Johnson and Biopsys Medical, Inc. Complete Merger. http://prnewswire.com/cgi-bin/stories.pl?ACCT=104&STORY=/www/story/7-31-97/289165&EDATE=.

[B4] Burbank F, Parker SH, Fogarty TJ (1996). Stereotactic breast biopsy: improved tissue harvesting with the Mammotome. Am Surg.

[B5] Parker SH, Dennis MA, Stavros AT, Johnson KK (1996). Ultrasound-Guided Mamtmeotomoy: A New Breast Biopsy Technique. J Diagn Med Sonography.

[B6] Scheler P, Pollow B, Hahn M, Kuner RP, Fischer A, Hoffmann G (2000). [Hand-held ultrasound-guided vacuum biopsy of mammary lesions–first experiences]. Zentralbl Gynakol.

[B7] Simon JR, Kalbhen CL, Cooper RA, Flisak ME (2000). Accuracy and complication rates of US-guided vacuum-assisted core breast biopsy: initial results. Radiology.

[B8] Fine RE, Israel PZ, Walker LC, Corgan KR, Greenwald LV, Berenson JE, Boyd BA, Oliver MK, McClure T, Elberfeld J (2001). A prospective study of the removal rate of imaged breast lesions by an 11-gauge vacuum-assisted biopsy probe system. Am J Surg.

[B9] Hung WK, Lam HS, Lau Y, Chan CM, Yip AW (2001). Diagnostic accuracy of vacuum-assisted biopsy device for image-detected breast lesions. ANZ J Surg.

[B10] Meloni GB, Dessole S, Becchere MP, Soro D, Capobianco G, Ambrosini G, Nardelli GB, Canalis GC (2001). Ultrasound-guided mammotome vacuum biopsy for the diagnosis of impalpable breast lesions. Ultrasound Obstet Gyneco.

[B11] Parker SH, Klaus AJ, McWey PJ, Schilling KJ, Cupples TE, Duchesne N, Guenin MA, Harness JK (2001). Sonographically guided directional vacuum-assisted breast biopsy using a handheld device. AJR Am J Roentgenol.

[B12] Perez-Fuentes JA, Longobardi IR, Acosta VF, Marin CE, Liberman L (2001). Sonographically guided directional vacuum-assisted breast biopsy: preliminary experience in Venezuela. AJR Am J Roentgenol.

[B13] Fine RE, Boyd BA, Whitworth PW, Kim JA, Harness JK, Burak WE (2002). Percutaneous removal of benign breast masses using a vacuum-assisted hand-held device with ultrasound guidance. Am J Surg.

[B14] Johnson AT, Henry-Tillman RS, Smith LF, Harshfield D, Korourian S, Brown H, Lane S, Colvert M, Klimberg VS (2002). Percutaneous excisional breast biopsy. Am J Surg.

[B15] Chen SC, Yang HR, Hwang TL, Chen MF, Cheung YC, Hsueh S (2003). Intraoperative ultrasonographically guided excisional biopsy or vacuum-assisted core needle biopsy for nonpalpable breast lesions. Ann Surg.

[B16] Fine RE, Whitworth PW, Kim JA, Harness JK, Boyd BA, Burak WE (2003). Low-risk palpable breast masses removed using a vacuum-assisted hand-held device. Am J Surg.

[B17] Huber S, Wagner M, Medl M, Czembirek H (2003). Benign breast lesions: minimally invasive vacuum-assisted biopsy with 11-gauge needles patient acceptance and effect on follow-up imaging findings. Radiology.

[B18] March DE, Coughlin BF, Barham RB, Goulart RA, Klein SV, Bur ME, Frank JL, Makari-Judson G (2003). Breast masses: removal of all US evidence during biopsy by using a handheld vacuum-assisted device–initial experience. Radiology.

[B19] Philpotts LE, Hooley RJ, Lee CH (2003). Comparison of automated versus vacuum-assisted biopsy methods for sonographically guided core biopsy of the breast. AJR Am J Roentgenol.

[B20] Sperber F, Blank A, Metser U, Flusser G, Klausner JM, Lev-Chelouche D (2003). Diagnosis and treatment of breast fibroadenomas by ultrasound-guided vacuum-assisted biopsy. Arch Surg.

[B21] Alonso-Bartolome P, Vega-Bolivar A, Torres-Tabanera M, Ortega E, Acebal-Blanco M, Garijo-Ayensa F, Rodrigo I, Munoz-Cacho P (2004). Sonographically guided 11-G directional vacuum-assisted breast biopsy as an alternative to surgical excision: utility and cost study in probably benign lesions. Acta Radiol.

[B22] Semiz Oysu A, Kaya H, Gulluoglu B, Aribal E (2004). Comparison of sonographically guided vacuum-assisted and automated core-needle breast biopsy methods. Tani Girisim Radyol.

[B23] Carpentier E, Maruani A, Michenet P, Bonneau C, Degand P, Lebas P (2005). Can US-guided vacuum-assisted biopsies be an alternative to diagnostic surgery in cases of non-diagnostic core needle biopsy?. J Radiol.

[B24] Ceccarelli G, Casciola L, Battistini I, Stefanoni M, Spaziani A, Conti D, Di Zitti L, Valeri R, Bartoli A, Bellochi R, Rambotti M, Pisanelli MC (2005). Non palpable lesions of the breast: the Mammotome-biopsy in the preoperative management of breast cancer. G Chir.

[B25] Cho N, Moon WK, Cha JH, Kim SM, Kim SJ, Lee SH, Chung HK, Cho KS, Park IA, Noh DY (2005). Sonographically guided core biopsy of the breast: comparison of 14-gauge automated gun and 11-gauge directional vacuum-assisted biopsy methods. Korean J Radiol.

[B26] Costantini R, Sardellone A, Marino C, Giamberardino MA, Innocenti P, Napolitano AM (2005). Vacuum-assisted core biopsy (Mammotome) for the diagnosis of non-palpable breast lesions: four-year experience in an Italian center. Tumori.

[B27] Grady I, Gorsuch H, Wilburn-Bailey S (2005). Ultrasound-guided, vacuum-assisted, percutaneous excision of breast lesions: an accurate technique in the diagnosis of atypical ductal hyperplasia. J Am Coll Surg.

[B28] Plantade R, Hammou JC, Gerard F, Chanalet I, Aubanel D, David-Bureau M, Scotto A, Fighiera M, Gueret S, Lo Monaco L (2005). [Ultrasound-guided vacuum-assisted biopsy: review of 382 cases]. J Radiol.

[B29] Cassano E, Urban LA, Pizzamiglio M, Abbate F, Maisonneuve P, Renne G, Viale G, Bellomi M (2007). Ultrasound-guided vacuum-assisted core breast biopsy: experience with 406 cases. Breast Cancer Res Treat.

[B30] Govindarajulu S, Narreddy SR, Shere MH, Ibrahim NB, Sahu AK, Cawthorn SJ (2007). Sonographically guided mammotome excision of ducts in the diagnosis and management of single duct nipple discharge. Eur J Surg Oncol.

[B31] Sebag P, Tourasse C, Rouyer N, Lebas P, Denier JF, Michenet P (2006). [Value of vacuum assisted biopsies under sonography guidance: results from a multicentric study of 650 lesions]. J Radiol.

[B32] Vargas HI, Vargas MP, Gonzalez K, Burla M, Khalkhali I (2006). Percutaneous excisional biopsy of palpable breast masses under ultrasound visualization. Breast J.

[B33] Xiao L, Zhou P, Li RZ, Zhu WH, Wu JH (2006). Comparison of ultrasound-guided mammotome and Tru-cut biopsy needle in diagnosing breast masses. Zhong Nan Da Xue Xue Bao Yi Xue Ban.

[B34] NeuroScience Associates: Alternative Means for Calculating Volumes in Tissue Sections. http://www.neuroscienceassociates.com/Documents/Publications/volumetric.htm.

[B35] Quick Reference For: Mammotome^® ^Probe Specifications (Ultrasound). Ethicon Endo-Surgery, Inc, DSL# 05-0307.

[B36] Smith LF, Henry-Tillman R, Harms S, Hronas T, Mancino AT, Westbrook KC, Korourian S, Jones MP, Klimberg VS (2001). Hematoma-directed ultrasound-guided breast biopsy. Ann Surg.

[B37] Thompson M, Henry-Tillman R, Margulies A, Thostenson J, Bryant-Smith G, Fincher R, Korourian S, Klimberg VS (2007). Hematoma-directed ultrasound-guided (HUG) breast lumpectomy. Ann Surg Oncol.

[B38] Dash N, Chafin SH, Johnson RR, Contractor FM (1999). Usefulness of tissue marker clips in patients undergoing neoadjuvant chemotherapy for breast cancer. AJR Am J Roentgenol.

[B39] Baron LF, Baron PL, Ackerman SJ, Durden DD, Pope TL (2000). Sonographically guided clip placement facilitates localization of breast cancer after neoadjuvant chemotherapy. AJR Am J Roentgenol.

[B40] Schulz-Wendtland R, Heywang-Kobrunner SH, Aichinger U, Kramer S, Wenkel E, Bautz W (2002). [Do tissue marker clips after sonographically or stereotactically guided breast biopsy improve follow-up of small breast lesions and localisation of breast cancer after chemotherapy?]. Rofo.

